# 1,4-Butanediol – Determination of 1,4-butanediol in workplace air using gas chromatography (GC-FID): Air Monitoring Method – Translation of the German version from 2025

**DOI:** 10.34865/am11063e10_3or

**Published:** 2025-09-29

**Authors:** Silke Werner, Lutz Nitschke, Ralph Hebisch, Uta Lewin-Kretzschmar, Andrea Hartwig, MAK Commission

**Affiliations:** 1 Institute for Occupational Safety and Health of the DGUV (IFA). German Social Accident Insurance (DGUV) Alte Heerstraße 111 53757 Sankt Augustin Germany; 2 Bavarian State Office for Health and Food Safety (LGL) Pfarrstraße 3 80538 München Germany; 3 Federal Institute for Occupational Safety and Health (BAuA) Friedrich-Henkel-Weg 1–25 44149 Dortmund Germany; 4 German Social Accident Insurance, Institution for the raw materials and chemical industry, Prevention – Department of Hazardous Substances, Biological Agents and Analytical Chemistry Kurfürsten-Anlage 62 69115 Heidelberg Germany; 5 Institute of Applied Biosciences. Department of Food Chemistry and Toxicology. Karlsruhe Institute of Technology (KIT) Adenauerring 20a, Building 50.41 76131 Karlsruhe Germany; 6 Permanent Senate Commission for the Investigation of Health Hazards of Chemical Compounds in the Work Area. Deutsche Forschungsgemeinschaft, Kennedyallee 40, 53175 Bonn, Germany. Further information: Permanent Senate Commission for the Investigation of Health Hazards of Chemical Compounds in the Work Area | DFG

**Keywords:** 1,4-butanediol, air analyses, analytical method, workplace measurement, hazardous substance, gas chromatography, flame ionisation detection, GC-FID, glass fibre filter, activated carbon

## Abstract

The working group “Air Analyses” of the German Senate Commission for the Investigation of Health Hazards of Chemical Compounds in the Work Area (MAK Commission) developed and verified the presented analytical method. It is used to determine the levels of 1,4-butanediol [110-63-4] in workplace air. The method covers concentrations in the range from one hundredth up to twice the current occupational exposure limit value (OELV) of 200 mg/m^3^. The method is also suitable for monitoring compliance with the short-term exposure limit (STEL; excursion factor 4) for the inhalable fraction and vapour. Samples are collected by drawing a defined volume of air through a glass fibre filter and a sampling tube filled with activated charcoal which are inserted in a GGP mini sampling system using a flow regulated pump at a volumetric flow rate of 0.333 l/min. Exposure during the shift is assessed with a sampling period of 2 hours and the short-term exposure with a period of 15 minutes. The 1,4-butanediol deposited on the glass fibre filter and adsorbed to the activated charcoal is extracted by liquid desorption with dichloromethane/methanol (7:3 (v/v)) and analysed by gas chromatography using flame ionisation detection. The quantitative determination is based on multiple-point calibrations with an internal standard. A relative limit of quantification (LOQ) of 2 mg/m^3^ is obtained for an air sample volume of 40 litres. As the LOQ for a sample volume of 5 litres is below 400 mg/m^3^, the STEL can also be measured. The recovery is 95–101% and the expanded uncertainty is below 29% for a sampling period of 2 hours.

**Table:** 

**Method number**	1
**Application**	Air analysis
**Analytical principle**	Gas chromatography with flame ionisation detection (GC-FID)

## 1 Characteristics of the method

**Table:** 

**Precision:**	Coefficient of variation:	*V_x_* = 1.8 to 6.5%
	Expanded uncertainty:	*U* = 28%
	in the concentration range c = 2 to 446 mg/m^3^ and for n = 6 determinations	
**Limit of quantification:**	15.3 µg/ml	
	2 mg/m^3^ for an air sample volume of 40 l and a sampling period of 2 h	
**Recovery:**	*η* = 0.95–1.01 (95–101%)	
**Sampling recommendations:**	Sampling period:	2 h
	Air sample volume:	40 l
	Volumetric flow rate:	0.333 l/min
	For short-term measurements:	15 min, 0.333 l/min

## 2 Description of the substance

### 1,4-Butanediol [110-63-4] 

**Fig. 1:**
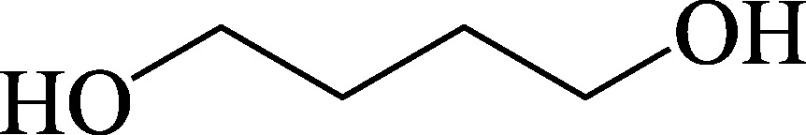
Structural formula of 1,4-butanediol

1,4-Butanediol (see Figure 1, also called tetramethylene glycol, 1,4-butylene glycol or 1,4-dihydroxybutane) is a colourless, odourless liquid (molar mass 90.12 g/mol, melting point 20 °C, boiling point 230 °C (IFA 2024), density at 25 °C 1.017 g/cm^3^ (Sigma-Aldrich 2024)). In the chemical industry, 1,4-butanediol is an important intermediate in the synthesis of other substances, e.g. tetrahydrofuran, and serves as the starting material for the production of a large number of secondary products such as polyesters, polyamides and polyurethanes. The substance is furthermore used as a solvent in paints, varnishes and toners (ECHA 2024; RÖMPP-Redaktion 2017).

An occupational exposure limit value (OELV) of 200 mg/m^3^ with an excursion factor of 4 has been established for 1,4-butanediol (AGS 2024). A MAK value has not been derived (DFG 2024). Due to its physicochemical properties, 1,4-butanediol occurs in the workplace air both in vapour and in particle form (DIN 2023).

## 3 General principles

This analytical method is used to determine 1,4-butanediol in the workplace air in a measurement range from 2 to about 446 mg/m^3^. This corresponds to one hundredth to twice the currently valid OELV.

A pump with a suitable capacity is used to draw a defined volume of air through a GGP mini sampling system equipped with a 13-mm glass fibre filter and an activated charcoal tube (type BIA) downstream of the filter. The 1,4-butanediol present in the air in gas or particle form is adsorbed to the sample carriers. For the analytical determination, the filter and activated charcoal tube are extracted together using dichloromethane/methanol (7:3 (v:v)). The analytes are separated by gas chromatography using a polar separation column and flame ionisation detection (GC-FID) for the qualitative and quantitative determination. The quantitative analysis is carried out using the internal standard method.

## 4 Equipment, chemicals and solutions

### 4.1 Equipment

For sampling:

- Pump for personal and stationary sampling, suitable for a volumetric flow rate of 0.333 l/min (e.g. GilAir Plus, supplied by DEHA Haan & Wittmer GmbH, 71296 Heimsheim, Germany)
- Sampling head for personal sampling of the inhalable fraction (GGP mini 0.33) (e.g. from GSA Messgerätebau GmbH, 40880 Ratingen, Germany)
- Glass fibre filter, Ø 13 mm (e.g. MN 85/90 BF, from Macherey und Nagel GmbH, 52355 Düren, Germany)
- Activated charcoal tubes, type BIA (e.g. from Dräger AG & Co. KGaA, 23560 Lübeck, Germany)
- Glass cutter
- Silicone tubing
- Silicone adapter GGP mini (e.g. from Carmacon, 67574 Osthofen, Germany)
- Flow meter (e.g. TSI Flowmeter 4146, from TSI GmbH, 52068 Aachen, Germany)

For sample preparation and the analytical determination:

- Screw-top vials with caps and septa, nominal volume 20 ml (e.g. from LABC Labortechnik Zillger KG, 53773 Hennef, Germany)
- Disposable tweezers (e.g. from LABC Labortechnik Zillger KG, 53773 Hennef, Germany)
- Volumetric flasks (glass), nominal volume 5 ml (e.g. from Brand GmbH + Co KG, 97877 Wertheim, Germany)
- Amber glass bottle 1000 ml (e.g. from Brand GmbH + Co KG, 97877 Wertheim, Germany)
- Graduated cylinder 1000 ml (e.g. from Brand GmbH + Co KG, 97877 Wertheim, Germany)
- Variable piston pipettes 10–100 µl and 100–1000 µl with pipette tips 100, 200 and 1000 µl (e.g. Eppendorf Multipette E3 with Combitips, from Eppendorf SE, 22366 Hamburg, Germany)
- Microlitre syringes, nominal volume 1 µl (e.g. from Hamilton Germany GmbH, 82166 Gräfelfing, Germany)
- Gas chromatograph with flame ionisation detector (FID) and data analysis programme (e.g. from PerkinElmer LAS GmbH, 63110 Rodgau, Germany)
- Disposable syringes, volume 2 ml, with disposable cannulas 0.9 × 40 mm (e.g. from B. Braun SE, 34212 Melsungen, Germany)
- Syringe filters with polytetrafluoroethylene membranes (PTFE) 13 mm, pore size 0.45 µm (e.g. VWR International GmbH, 64293 Darmstadt, Germany)
- Autosampler vials, nominal volume about 1.5 ml (e.g. LABC Labortechnik Zillger KG, 53773 Hennef, Germany)
- Screw caps for autosampler vials (e.g. CS-Chromatographie-Service GmbH, 52379 Langerwehe, Germany)
- Analytical balance, weighing range from 0.01 mg to 220 g, readability 0.01 mg (e.g. XP 205 Delta Range, from Mettler-Toledo GmbH, 35396 Gießen, Germany)
- Polar separation column (e.g. StabilWax, length (L) 60 m, inner diameter (ID) 0.25 mm, film thickness 0.5 µm, from Restek GmbH, 61348 Bad Homburg, Germany)

### 4.2 Chemicals

- Methanol for analysis, ≥ 99.9% (e.g. from Merck KGaA, 64293 Darmstadt, Germany)
- Dichloromethane for analysis, ≥ 99.8% (e.g. from Merck KGaA, 64293 Darmstadt, Germany)
- 1,4-Butanediol, 99% (e.g. ReagentPlus, from Sigma-Aldrich Chemie GmbH, 82024 Taufkirchen, Germany)
- 1-Hexanol for synthesis, ≥ 98.0% (e.g. from Merck KGaA, 64293 Darmstadt, Germany)
- Helium 5.0
- Hydrogen 5.0
- Synthetic air (hydrocarbon-free)

### 4.3 Solutions

The following solutions were prepared using the chemicals listed in Section 4.2:

**Extraction solution: **(dichloromethane/methanol, 7:3 (v:v))

To prepare the extraction solution, 700 ml dichloromethane and 300 ml methanol are measured separately into a 1000-ml graduated cylinder one after the other and then transferred into an amber glass bottle of suitable volume. The bottle is sealed and shaken.

The solvent mixture can be stored at room temperature and is stable for 4 weeks.

**Calibration stock solution:** (19.3 mg 1,4-butanediol/ml)

About 3 ml of extraction solution are placed into a 5-ml volumetric flask. 95 µl of 1,4-butanediol is dispensed into the volumetric flask using a piston pipette. The volumetric flask is filled to the mark with extraction solution and then shaken.

The calibration stock solution is transferred into a screw-top vial of suitable volume, sealed with a screw cap and labelled with the name of the solution and the date of preparation. The solution can be stored in the refrigerator for at least 6 months at 6 °C.

**Control stock solution:** (4.1 mg 1,4-butanediol/ml)

About 3 ml of extraction solution is placed into a 5-ml volumetric flask. 20 µl of 1,4-butanediol is dispensed into the volumetric flask using a piston pipette. The volumetric flask is filled to the mark with extraction solution and then shaken.

The control stock solution and the calibration stock solution are prepared separately.

The control stock solution is filled into a suitable screw-top vial, sealed with a screw cap and labelled with the name of the solution and the date of preparation. The solution can be stored in the refrigerator for at least 6 months at 6 °C.

### 4.4 Calibration standards

Five calibration solutions are prepared with dilutions of the calibration stock solution.

About 1.5 ml of extraction solution is placed into five 5-ml volumetric flasks each. Calibration stock solution is added in the volumes given in Table 1. The volumetric flasks are filled to the mark with extraction solution. 0.5 μl of 1-hexanol is added as the internal standard (ISTD) using a suitable microlitre syringe and the flasks are then shaken.

The calibration solutions are prepared fresh before every calibration.

**Tab. 1:** Preparation and concentrations of the calibration standards

Volume of the calibration stock solution [µl]	Mass concentration 1,4-butanediol [µg/ml]
4.0	15.4
20	77.3
40	155
70	271
150	580

### 4.5 Control standards

Control standards with concentrations in the lower range of the analytical procedure must be analysed every working day in duplicate under the conditions given in Section 6.

**Control standard: **(21.2 µg 1,4-butanediol/ml)

26 µl of the control stock solution is transferred to a 5-ml volumetric flask containing about 1.5 ml of extraction solution by piston pipette. The flask is filled to the mark with extraction solution, 0.5 µl of ISTD is added and the flask is shaken. The solution is filled into autosampler vials and stored in the refrigerator for daily monitoring every working day. If the concentration deviates by more than ± 10%, a new control standard is prepared that is then used to repeat the check.

## 5 Sampling and sample preparation

### 5.1 Sampling

Samples are collected using stationary or personal sampling procedures. The samples taken by personal sampling are collected within the breathing zone. The inlet of the sampling head must remain unobstructed during sampling.

The samples are taken with flow-regulated pumps of suitable capacity. Using a representative sample carrier to simulate upstream resistance, the volumetric flow rate is set to 20 l/h (0.333 l/min).

Immediately prior to sampling, a glass cutter is used to cut open the activated charcoal tube at both ends. One end of the activated charcoal tube is connected to the sampling head containing the glass fibre filter with a piece of tubing (silicone), keeping the direction of air flow in mind. The other end of the activated charcoal tube is connected to the pump in the direction of the arrow. The recommended sampling period is two hours. Sampling for two hours at a volumetric flow rate of 0.333 l/min is equivalent to an air sample volume of 40 l. The main parameters for determining concentrations in air (air sample volume, temperature, air pressure and relative humidity) are documented in a sampling record.

Immediately after sampling, the activated charcoal tube is sealed with suitable caps. The sampling head of the GGP mini is unscrewed. Tweezers are used to transfer the glass fibre filter into a prepared screw-top vial, which is then sealed airtight. The loaded sample carriers must be transported unopened to the laboratory, if possible within 7 days, and are stored there at room temperature until processing.

The flow rate must be checked for constancy after sampling. If the deviation from the adjusted flow rate is ≥ ± 5%, the measurement should be repeated.

### 5.2 Sample preparation

The activated charcoal tube is opened in the laboratory and the entire contents of the tube are transferred to the screw-top vial containing the respective glass fibre filter. The glass fibre filter and the activated charcoal are then covered with 5 ml of extraction solution. 0.5 µl of 1-hexanol is added as the ISTD. The vials are sealed and let stand overnight at room temperature. The extract is collected by disposable syringe and then passed through a syringe filter into an autosampler vial. The sample is analysed in duplicate by gas chromatography under the conditions given in Section 6. If the signal lies above the validated range of the analytical procedure, the sample is diluted and then analysed again.

## 6 Operating conditions

**Table:** 

**Apparatus:**	Gas chromatograph PerkinElmer 500, with FID and autosampler	
**Separation column:**	Material: Length: Inner diameter: Film thickness:	StabilWax 60 m 0.25 mm 0.5 µm
**Injection volume:**	1 µl	
**Injector temperature:**	200 °C	
**Detector:**	FID	
**Detector temperature:**	250 °C	
**Oven programme:**	Initial temperature 50 °C; maintain for 2.5 min increase by 20 °C/min to 200 °C; maintain for 10 min	
**Carrier gas:**	Helium 5.0	
**Carrier gas flow:**	280 kPa	
**Split:**	15 ml/min	

## 7 Analytical determination

An autosampler is used to inject 2 doses of 1 μl of the prepared samples into the gas chromatograph. The samples are analysed under the conditions described in Section 6.

If the concentrations that are determined are above the calibration range, a suitable dilution of the sample must be prepared with extraction solution and the analysis repeated.

## 8 Calibration

The calibration standards described in Section 4.4 are used to derive the calibration functions. Each of the calibration standards are injected in 3 doses of 1 μl and analysed as described in Sections 6 and 7. The quotients of the measured peak areas of 1,4-butanediol and ISTD are plotted against the quotients of the corresponding concentrations of 1,4-butanediol and ISTD. The calibration function is linear in the investigated concentration range.

A control standard must be analysed every working day to check the calibration function (see Section 4.5). A new calibration must be performed if the analytical conditions change or the results of the quality control indicate that this is necessary.

## 9 Calculation of the analytical result

Equation 1 is used to calculate the concentration in the extraction solution based on the mass of 1,4-butanediol per sample carrier determined by the data analysis programme:



(1)

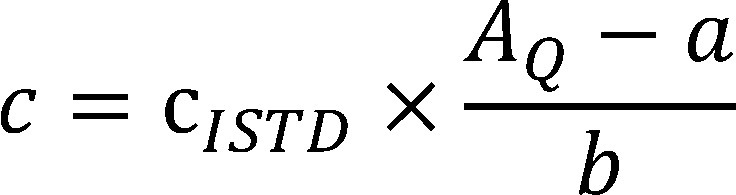




where:

**Table:** 

*c*	is the mass concentration of the substance in the extraction solution in mg/l
*c_ISTD_*	is the mass concentration of the ISTD in the extraction solution in mg/l
*A_Q_*	is the peak area quotient 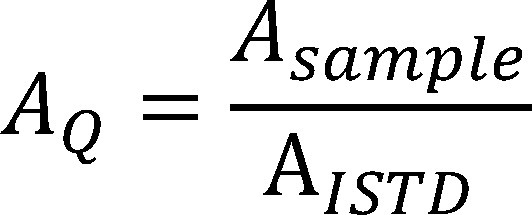
*a*	is the y-intercept
*b*	is the slope of the graph

This value is used to calculate the concentration in the workplace air according to Equation 2, taking into consideration the extraction volume, the air sample volume and the recovery:



(2)

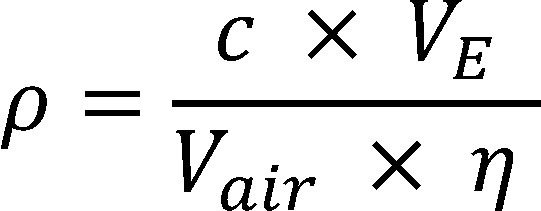




where:

**Table:** 

*ρ*	is the mass concentration of the substance in the air sample in mg/m^3^
*V_E_*	is the volume of the extraction solution in l (in this case 0.005 l)
*V_air_*	is the air sample volume in m^3^ (determined based on the volumetric flow rate and the sampling period, in this case 0.04 m^3^ after a sampling period of 2 hours)
*ƞ*	is the recovery

## 10 Reliability of the method

The characteristics of the method were determined according to the standards DIN EN 482 (DIN 2021), ISO 20581 (DIN 2016), DIN 32654 (DIN 2008) and DIN EN ISO 23861 (DIN 2023).

To determine the characteristics of the method, the sample carriers were prepared as described in Section 5.1. The glass fibre filters were spiked with respective solutions of 1,4-butanediol. The spiked amounts of 1,4-butanediol were in the range of 0.08 mg to 17.8 mg per sample carrier. Assuming an air sample volume of 40 l, this is equivalent to a concentration range of 2.0 mg/m^3^ to 446 mg/m^3^. Conditioned air from a dynamic test gas facility was drawn through the spiked sample carriers for 2 hours at a volumetric flow rate of 0.333 l/min.

### 10.1 Precision, recovery and expanded uncertainty

The precision and recovery experiments were carried out at a relative humidity of about 40%. The samples were analysed as described in Sections 5 and 6.

To determine recovery, sets of 6 sample carriers were loaded per experiment. Table 2 shows the coefficients of variation and percent recovery determined from these samples and the expanded uncertainties calculated from these values. The calculation was carried out according to DIN EN ISO 23861 (DIN 2023) using the IFA service tool for calculating expanded measurement uncertainty (IFA n.d.).

**Tab. 2:** Characteristics of the method and expanded uncertainty

Concentration^a)^ [mg/m^3^]	Recovery [%]	Coefficient of variation [%]	Expanded uncertainty *U* [%]
2.0	95	5.1	28.3
20	100	6.5	28.3
91	98	6.5	28.4
202	100	4.7	28.4
446	100	1.8	27.9

^a)^ The concentration is calculated for a sampling period of 2 hours and a volumetric flow rate of 0.333 l/min.

The expanded uncertainty was determined after taking all relevant influencing parameters into consideration. The two main sources of uncertainty are uncertainties in the sampling procedure and those in the analytical procedure.

The combined, concentration-dependent uncertainties for the entire method were calculated by combining the contributions from all sources of uncertainty. The percentages listed in Table 2 for the expanded uncertainties for the entire method were obtained by multiplying these values with the expansion factor k = 2.

### 10.2 Limit of quantification

The limit of quantification (LOQ) was determined according to the blank value method described in DIN 32654 (DIN 2008). The limits of quantification are 15.3 µg/ml absolute or 2 mg/m^3^ for an air sample volume of 40 litres (0.333 l/min and a sampling period of 2 h).

### 10.3 Influence of humidity

Samples were collected at a relative humidity of about 20% and about 80%. Sets of 7 sample carriers were loaded for each concentration tested (LOQ, 0.1 OELV, 1 OELV and 2 OELV) as described in Section 10.

Humidity was found to influence the recovery in the range of the LOQ at about 20% humidity. The percent recovery was 94.6% in the range of the LOQ. In this case, the measured value must be limited or corrected accordingly.

The influence of humidity in the range between 0.01 OELV and 0.1 OELV must be assessed separately. Humidity was not found to influence the recovery at concentrations of 0.1 OELV to 2 OELV.

### 10.4 Influence of temperature

The influence of temperature was investigated by collecting samples at temperatures of about 10 °C and of about 40 °C. For this purpose, sets of 7 sample carriers per test concentration (LOQ, 0.1 OELV and 2 OELV) were prepared as described in Section 10. The sample carriers were stored in a climate chamber; air was drawn through the sample carriers in the chamber for 2 hours using a suitable pump at a volumetric flow rate of 0.333 l/min.

The temperature had an influence on recovery in the examined concentration range at the LOQ at a temperature of about 10 °C. A recovery of 72% was determined under the described conditions. In this case, the measured values must be corrected accordingly. The temperature did not have an influence on the recovery at concentrations of 0.1 OELV to 2 OELV.

### 10.5 Capacity of the sampling system

To determine the breakthrough behaviour of the sampling system used for testing, the GGP mini, equipped with a glass fibre filter and an activated charcoal tube, was connected to a second activated charcoal tube by tubing. The glass fibre filter was spiked with 25.9 mg of 1,4-butanediol, which is equivalent to a concentration in air of 433 mg/m^3^ after sampling for 3 hours. Conditioned air (0.333 l/min) from a dynamic test gas facility was drawn through the set-up for 3 hours (recommended sampling period plus one hour). The filter and the contents of the first activated charcoal tube were then transferred together to a screw-top vial and the contents of the second tube to a separate vial. The samples were prepared and analysed as described in Sections 5.2, 6 and 7.

A recovery of 99% was determined for the 1,4-butanediol adsorbed to the glass fibre filter and the first activated charcoal tube. 1,4-Butanediol was not detected on the second tube that had been connected downstream. Therefore, the sampling system is suitable for use with concentrations up to 433 mg/m^3^ and a sampling period of 3 hours.

### 10.6 Storage stability

The storage stability of the loaded sample carriers was determined by spiking sets of 6 sample carriers with concentrations of 2.0 mg/m^3^, 20.3 mg/m^3^ and 406 mg/m^3^ as described in Section 10. The samples were conditioned for 2 hours with air at high humidity (about 80%) and a volumetric flow rate of 0.333 l/min. The filters were removed from the GGP mini and transferred to a screw-top vial with tweezers. The vials were sealed and stored at room temperature. The activated charcoal tubes were sealed with caps and likewise stored at room temperature. The filters and the charcoal tubes were prepared and analysed as described in Sections 5.2, 6 and 7 after storage periods of 1 day, 3 days, and 1, 2, 3 and 4 weeks.

The loaded sample carriers have a storage stability of at most 7 days.

### 10.7 Selectivity

All materials and reagents must be checked for blank values, particularly when a new batch is used. No blank values were detected during the development of the method. Therefore, correction was not required.

**Fig. 2:**
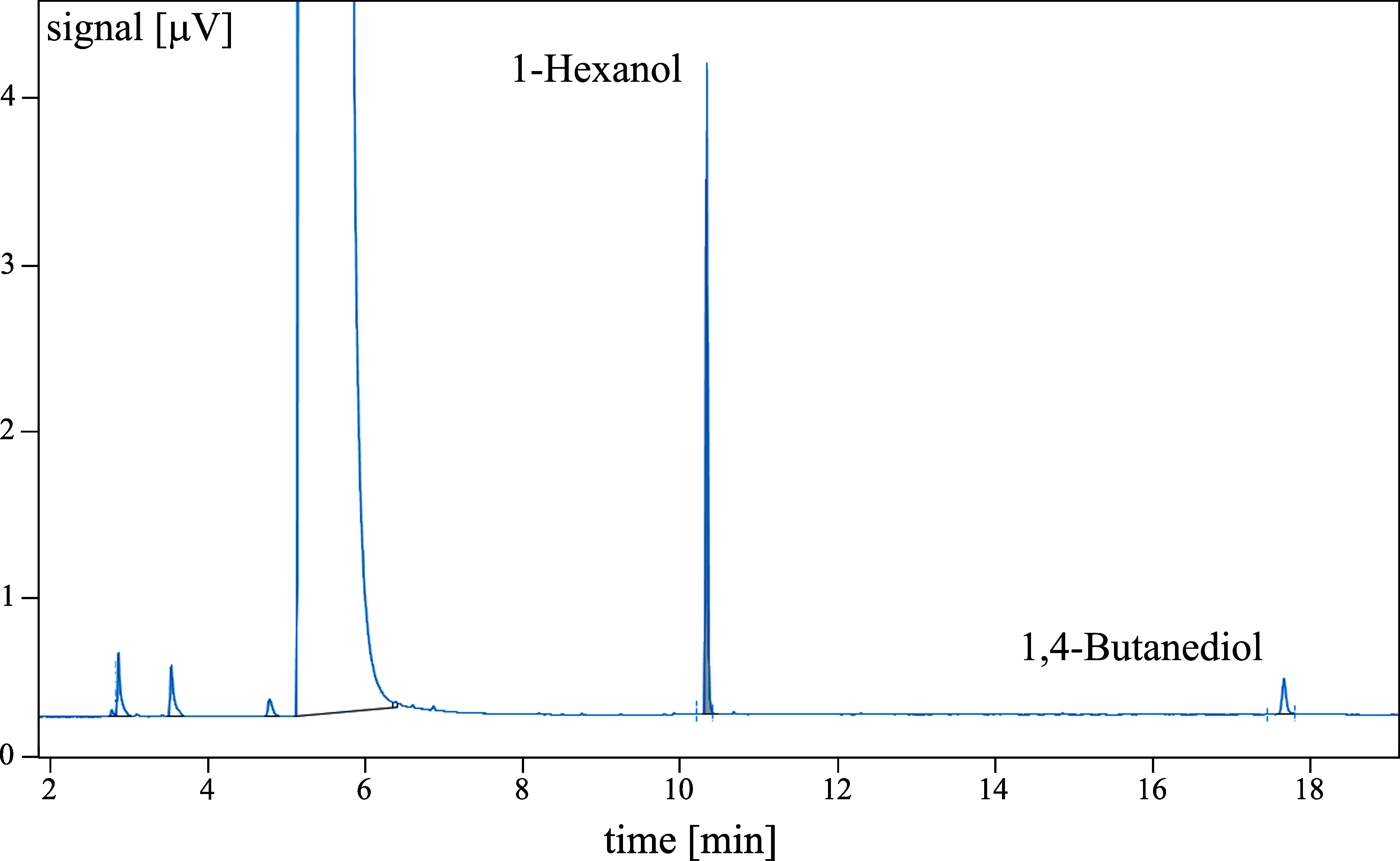
Gas chromatogram of 1,4-butanediol with the ISTD 1-hexanol

The determination of 1,4-butanediol is selective under the given conditions. The signals of the analyte and ISTD were clearly distinguishable (see Figure 2). However, if a signal with the same retention time as that of the analyte or ISTD is detected, this must be examined in more detail and the blank value must be considered when performing the analytical calculations for the samples.

## 11 Discussion

The analytical method described here is used to determine 1,4-butanediol in the workplace air in a concentration range from one hundredth to twice the currently valid OELV of 200 mg/m^3^. The method is also suitable for monitoring compliance with the STEL.
